# Supplementary material for: Body Composition Assessment in Mexican Children and Adolescents. Part 1: Comparisons between Skinfold-Thickness, Dual X-ray Absorptiometry, Air-Displacement Plethysmography, Deuterium Oxide Dilution, and Magnetic Resonance Imaging with the 4-C Model
Source: Nutrients. 2022 Mar 3;14(5):1073. doi: 10.3390/nu14051073 (PMC8912681; doi:10.3390/nu14051073)

Body composition assessment in Mexican children and adolescents. Part 1: comparisons between skinfold-thickness, dual x-ray absorptiometry, air-displacement pletismography, deuterium oxide dilution, magnetic resonance imaging with the 4-C model.

SUPPLEMENTARY MATERIALS.

Supplementary Figure S1. Differential correlation between methods (SF, DXA, ADP, D2O and MRI vs 4C), across levels of FM at left and FFM at right.

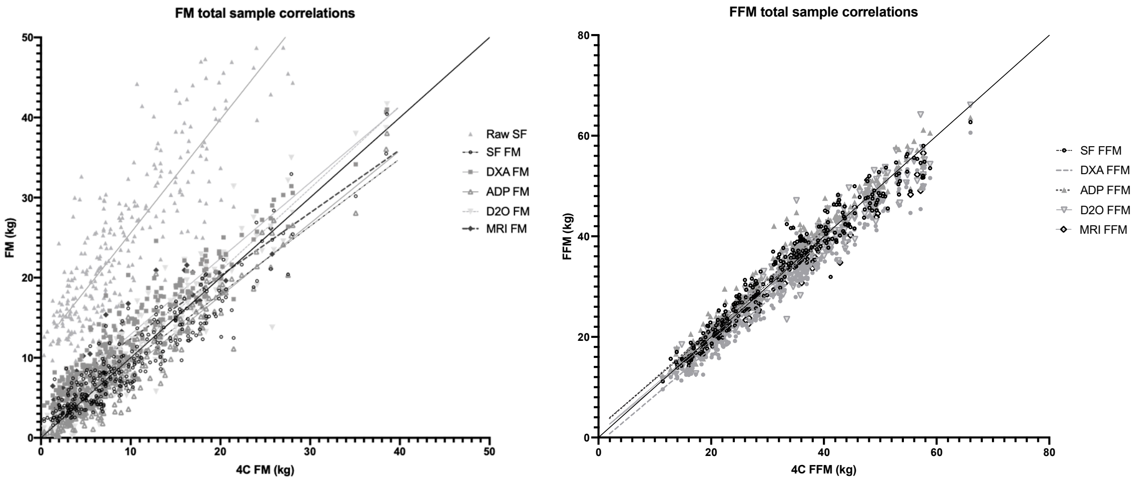

Supplementary Table S1. Demographic and clinical characteristics data of MRI subsample (n = 52).

Male	28 (54%)
Age (years)	12.6 ± 3.7
Weight (kg)	46.0 ± 15.8
Weight z-score	-0.21 ± 0.97
Height (cm)	123.5 ± 14.7
Height z-score	0.11 ± 1.07
BMI (kg/m2)	17.1 ± 6.0
BMI z-score	0.11 ± 1.07
Waist circumference (cm)	68.6 ± 12.0
Tanner	
1	14 (78%)
2	5 (10%)
3	20 (38%)
4	11 (21%)
5	2 (4%)
BMI category	
Healthy weight	39 (74%)
Overweight	13 (25%)
Body composition variables	
SF-FM (kg)	10.0 ± 5.8
D_2O FM (kg)	10.9 ± 7.0
DXA FM (kg)	14.0 ± 6.8
ADP FM (kg)	9.9 ± 6.3
4C FM (kg)	10.6 ± 6.6
MRI FM (kg)	12.9 ± 5.7

BMI = body mass index, SF = skinfold-thickness, FM = fat mass, D2O= Deuterium oxide dilution, DXA= Dual x-ray absorptiometry, ADP= Air-displacement plethysmography, 4C = four compartment model, MRI= Magnetic Resonance Imaging.

Supplementary Table S2. Comparison of the FM means estimated by SF, ADP, DXA, D2O, MRI and contrasted with the 4-C model as reference standard by sex and age group.

Age group	Sex	4C model FM (kg)	SF FM (kg)		ADP FM (kg)		DXA FM (kg)		D_2O FM (kg)		MRI FM (kg)*	
		Mean ± SD	Mean ± SD	*P value*	Mean ± SD	*P value*	Mean ± SD	*P value*	Mean ± SD	*P value*	Mean ± SD	*P value*
Children (4-10 y)	Female n = 63	5.5 ± 4.2	5.0 ± 3.3	0.016	4.1 ± 4	< 0.001	8.2 ± 4.2	< 0.001	5.4 ± 4.5	0.723	8.8 ± 5.5	0.006
	Male n = 54	5.8 ± 4.3	5.5 ± 3.4	0.267	5.2 ± 4.2	0.002	8.3 ± 4.2	< 0.001	5.5 ± 4.4	0.17	7.0 ± 4.2	0.005
Adolescents (11-18 y)	Female n = 92	14.4 ± 7.3	13.5 ± 7.2	0.009	12.2 ± 6.9	< 0.001	17.5 ± 6.7	< 0.001	15.2 ± 8.1	0.005	16.4 ± 4.8	0.068
	Male n = 79	9.3 ± 5.8	9.7 ± 6.0	0.012	8.3 ± 5.9	0.001	12.5 ± 5.8	< 0.001	10.3 ± 6.4	0.007	12.6 ± 4.8	0.001
Total sample	n = 288	9.5 ± 6.8	9.1 ± 6.5	0.001	8.2 ± 6.5	< 0.001	12.5 ± 6.8	< 0.001	10.1 ± 7.4	< 0.001	12.9 ± 5.7	< 0.001

T paired test for Mean differences. *MRI data represents a subsample of 52 participants.

Supplementary Figure S2. Bland-Altman plots for FM% estimation by SF, DXA, ADP, D2O, and MRI in contrast with the 4-C model for all ages and sex groups.

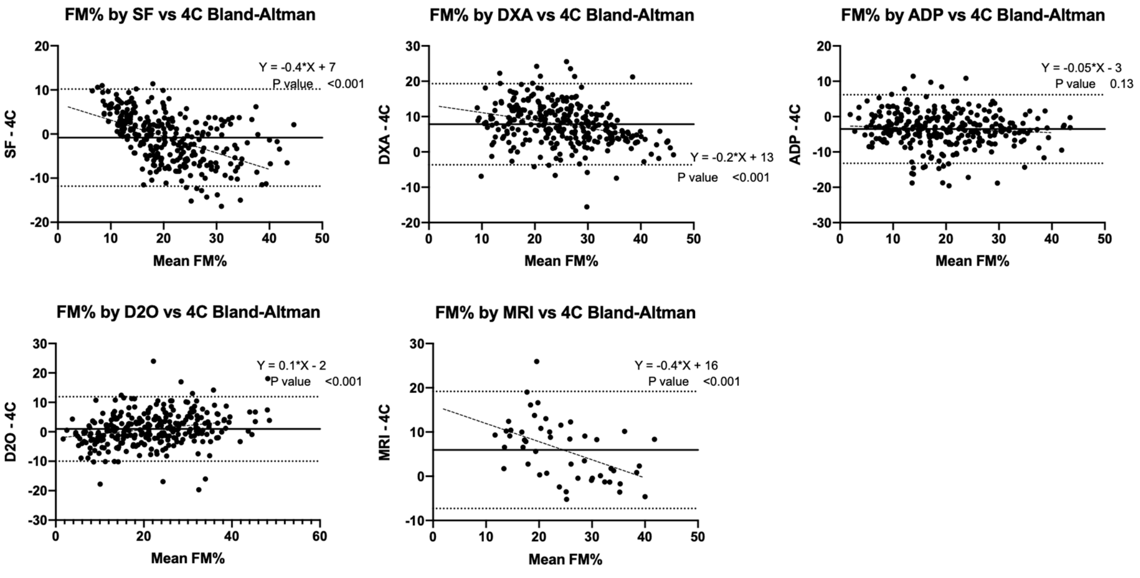

Supplementary Table S3. Agreement of FM estimation between the different methodologies.

	Method of BC measurement	n	Pearson correlation (95% CI)	Lin concordance coefficient (95% CI)	Bland-Altman Mean Difference ± SD (LOA)	Regression analysis from Bland-Altman differences
SF	DXA	293	0.97<br>(0.96 to 0.97)	0.85<br>(0.82 to 0.87)	3.23 ± 1.7<br>(-0.2 to 6.6)	Y = 0.09028X + 2.260<br><0.001
	ADP	293	0.95<br>(0.94 to 0.96)	0.94<br>(0.92 to 0.95)	-1.05 ± 2.0<br>(-5.1 to 3.4)	Y = -0.04195X - 1.409<br>0.02
	D2O	293	0.94<br>(0.93 to 0.96)	0.87<br>(0.84 to 0.89)	0.75 ± 3.5<br>(-6.2 to 7.7)	Y = 0.2008X - 1.159<br><0.001
	MRI	50	0.96<br>(0.93 to 0.97)	0.84<br>(0.76 to 0.89)	-2.59 ± 1.78<br>(-0.9 to 6.1)	Y = 0.0848X + 1.658<br>0.06
DXA	ADP	293	0.98<br>(0.97 to 0.98)	0.8<br>(0.77 to 0.83)	-4.28 ± 1.5<br>(-7.2 to 1.4)	Y = -0.04852X - 3.781<br><0.001
	D2O	293	0.89<br>(0.87 to 0.91)	0.84<br>(0.80 to 0.87)	-2.48 ± 3.4<br>(-9.2 to 4.2)	Y = 0.1066X - 3.666<br><0.001
	MRI	50	0.96<br>(0.94 to 0.98)	0.94<br>(0.91 to 0.96)	-1.07 ± 2.0<br>(-5.0 to 2.9)	Y = -0.1534X + 0.9016<br><0.001
ADP	D2O	293	0.87<br>(0.84 to 0.90)	0.84<br>(0.80 to 0.87)	1.79 ± 3.7<br>(-5.5 to 9.0)	Y = 0.1589X + 0.707<br><0.001
	MRI	50	0.94<br>(0.9 to 0.97)	0.82<br>(0.73 to 0.88)	2.93 ± 2.2<br>(-1.4 to 7.3)	Y = -0.08043X + 3.808<br>0.12
D2O	MRI	50	0.83<br>(0.72 to 0.9)	0.78<br>(0.67 to 0.86)	1.99 ± 4.1<br>(-6.0 to 10.0)	Y = -0.265X + 4.455<br>0.02

*LOA: limits of agreement

Supplementary Figure S3. Bland-Altman plots for FM estimated between the different methods: SF, DXA, ADP, D2O, and MRI for the total sample.

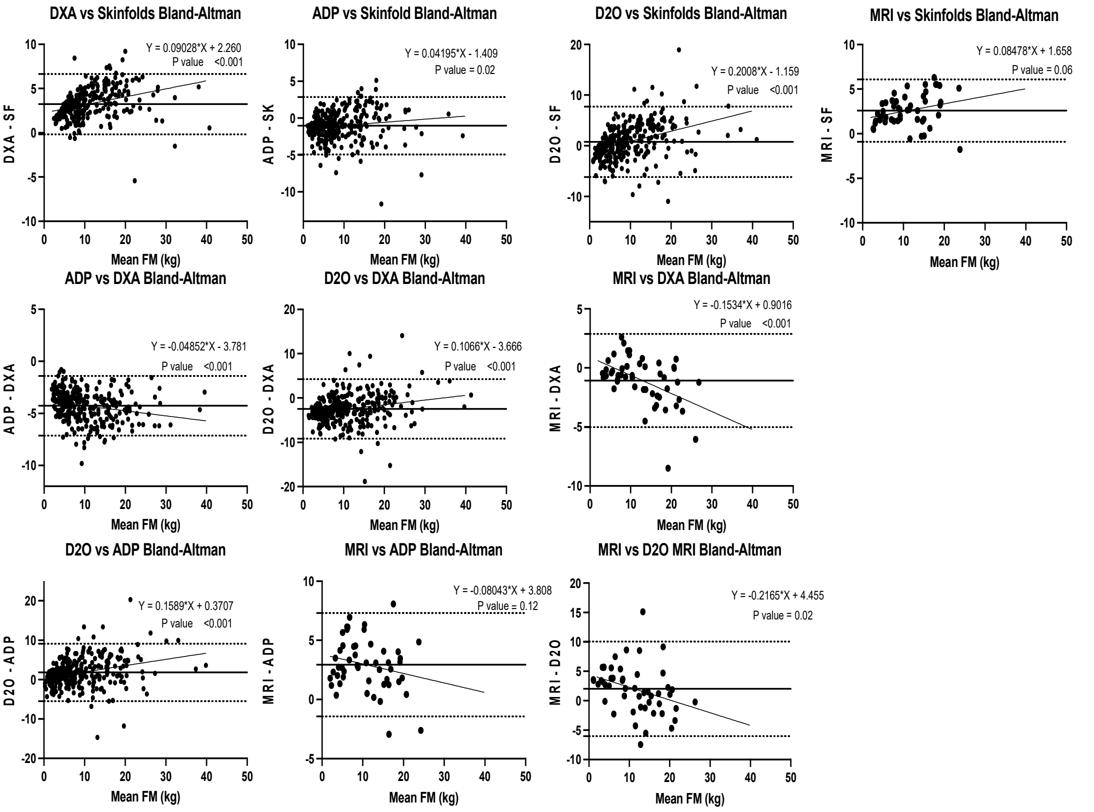

Supplement: Supplementary file 1 [file nutrients-14-01073-s001.zip › nutrients-1601338-supplementary.pdf]
